# 

**Published:** 1897-01-02

**Authors:** 


### Books Received.

George Newnes (Limited).

"The Story of the Chemical Elements." By Pattison Muir, M.A.

MacMillan and Co.

"Hygiene for Beginners." By E. S. Reynolds, M.D.

Adam and C. Black.

"Black's Guide to Bournemouth." By Hope Moncrieff.

The Rebman Publishing Company.

"Sanatoria for Consumptives." By Van Jaruntowsky, translated by Dr. Clifford Beale.

G. P. Johnston.

"The Use of Tuberculin." James Wilson, M.A., B.Sc.

**Periodicals.**—Humanitarian, Industries and Iron, British Review, Literary Digest, The Sun, London, Electrical Review, Nursing Notes, Invention.

---

**Notices of Births, Deaths, and Marriages** are inserted in this column at a charge of 2s. 6d. for an announcement not exceeding four lines.

### NOTICE TO CORRESPONDENTS.

All MS., Letters, Books for Review, and other matters intended for the Editor should be addressed The Editor, "The Hospital," The Hospital Building, 28 & 29, Southampton Street, Strand, W.C.

The Editor cannot undertake to return rejected MS., even when accompanied by stamped directed envelope.

**Notice to Advertisers and Subscribers.**

All Advertisements, Orders for Copies of The Hospital, and Business Communications should be addressed to **THE MANAGER, (Not to The Editor),** The Hospital, The Hospital Building, 28 & 29, Southampton Street, Strand, London, W.C.

The Cost of Subscription, post free, is as follows:—Per week, 2½d.; per quarter, 2s. 8d.; per half-year, 5s. 3d.; per annum, 10s. 6d. Foreign:—Per annum, 15s. 2d.; payable, in all cases, in advance.

**NOTICE.—Vol. XX. of "The Hospital" (April to September, 1896), in handsome cloth binding, is now on sale at the Office of this Journal, price 7s. 6d. Cases for binding the Numbers contained in this Volume 1s. 6d. Index to Vol. XX., 2½d., post free.**

**The Monthly Part for January is now ready. Price 6d., by post 8d.**

### Scraps and Gleanings.

The net amount handed in to the Central Committee as the result of the London Lifeboat Saturday Fund was £3,503.

A dispatch from Madrid states that General Weyler has telegraphed that 100 army surgeons are urgently needed in Cuba.

The Dental Hospital of London, Leicester Square, are appealing for funds towards defraying the cost of the erection of their new hospital.

The memorial statue to Surgeon-Major Parke, erected by the medical profession in Ireland has been placed upon its pedestal at Leinster Lawn, Dublin.

The following extract from the nineteenth report of the Derbyshire Hospital for Sick Children is not as frank as it should be: "The total number of patients during the year has been 2,042, being an increase of 68 on last year, and being the largest number treated for several years, whilst the ordinary expenses are less than ever. Of the above number 222 were in-patients, and 1,820 out-patients." The suppression of the fact that the in-patients numbered 253 and the out-patients 1,721 in 1894-95 alters the suggestion conveyed in the coupling of the patients and expenditure in the first sentence. Economically managed though this hospital is, the saving on the cost of 31 in-patients more than makes up for the increased expenditure entailed by treating 99 out-patients!

### The Student of Medicine.

### APPOINTMENTS.

J. J. Clarke, M.B.Lond., F.R.C.S.Eng , appointed Assistant Surgeon to the City Orthopædic Hospital. H. S. Cooper, L.R.C.P.Lond., M.R.C.S.Eng., appointed Medical Officer of Health to the Brightlingsea Urban District. A. Crisp, M.R.C.S.-Eng., reappointed Medical Officer for the Horsey District of the Smallburgh Union. L. C. Edwards, M.B , C.M.Edin., appointed Junior House Surgeon to the Chesterfield and North Derbyshire Hospital. W. J. Forbes, M.B., appointed Medical Officer for the Scriven District of the Knaresborough Union. Dr. Lace, appointed Certifying Factory Surgeon for the Cleator District. Mr. W. S. Langworthy, appointed Medical Officer for the No. 4 District of the Plympton St. Mary Union. J. Morgan, F.R.C.S.Eng., L.R.C.P.Lond , reappointed Medical Officer of Health to the Langport Rural District Council. W. H. Packer, M.D.Brux., L.R.C.P.Lond., M.R.C.S.Eng., appointed Medical Officer to the Atcham Workhouse Infirmary, Shrewsbury. Mr. W. R. Thompson, appointed Medical Officer of Health to the Shelf Urban District. T. J. Tubb, L.R.C.P.&S., D P.H.Irel., reappointed Medical Officer of Health for the Borough of Lowestoft and the Port Sanitary Authority; also Physician to the Borough Sanatorium, Lowestoft. T. Watts, M.B., B.S.Durh , M.R.C.S.Eng., L.R.C P.Lond., appointed Certifying Factory Surgeon for Hyde. C. W. Williams, M.R C S., L R C.P., L.S.A., appointed Surgeon to the Workhouse and Medical Officer for the Smallburgh District of the Smallburgh Union.

### PASS LISTS.

**University of London, 1896.**

M.D. Examination.

Medicine.—E. B. Allan, University College and University of Melbourne; H. R. Andrews, London Hospital; G. H. Barker, B.Sc., University College, Bristol; S. M. Brown, Owens College and Manchester Royal Infirmary; J. A. Codd, B.Sc., Yorkshire College; J. S. Collier, B.Sc., St. Mary's Hospital; F. G. Crookshank, University College; E. M. Cuffe, St. Bartholomew's Hospital; W. Edgecombe, University Colleges of Liverpool and London; L. E. V. Every-Clayton, B.S., Guy's Hospital; H. G. Felkin, London Hospital; S. E. Gill, St. Bartholomew's Hospital; J. H. Griffiths, B.S., St. Bartholomew's Hospital; E. M. Hainworth, B.S., B.Sc., St. Thomas's Hospital; W. S. Handley, B.S., Guy's Hospital; J. O. Harvey, St. Bartholomew's Hospital; R. Hopton, B.S., Yorkshire College; G. S. Hovenden, B.S., Guy's Hospital; A. W. Kirkpatrick-Picard, University College; W. E. Lee, St. Bartholomew's Hospital; A. G. Levy, University College; W. B. Morton, University College; F. L. Orr, University College; L. A. Parry, B.S., Guy's Hospital; C. H. Perram, St. Bartholomew's Hospital; F. J. Poynton, St. Mary's Hospital; M. H. Raper, Middlesex Hospital; E. N. Reichardt, St. Bartholomew's Hospital; A. J. Rodocanachi, B.S., B.Sc., University College; A. Salter, B.S., (Gold Medal), Guy's Hospital; E. W. Selby, B.S., University College; W. Shears, St. Bartholomew's Hospital; E. J. Smyth, B.S., B.Sc., University College; R. H. Steen, St. Mary's Hospital; G. H. C. Way, B.S., University College.

M.S. Examination.

H. A. Ballance, M.D., University College; H. L. Barnard, London Hospital; J. St. T. Clarke, M.D., Guy's Hospital; G. H. Cowen, London Hospital; J. W. F. Jewell, M.D., Guy's Hospital; W. P. Purvis, M.D., B.Sc., St. Thomas's Hospital; Mary Ann D. Scharlieb, M.D., London School of Medicine for Women; T. M. Thomas, Guy's Hospital.

B.S. Examination.

First Division.—J. H. Cook, University College: E. G. D. Drury, St. Bartholomew's Hospital; J. H. Hugo, St. Bartholomew's Hospital; A. W. Jenkins, University College; H. M. Rigby, London Hospital; H. J. Scharlieb, University College; Ethel M. Vaughan, London School of Medicine and Royal Free Hospital.

Second Division.—W. Allport, Queen's College and General Hospital, Birmingham; E. D. Aubin, Middlesex Hospital; C. Banting, University College; C. Bolton, B.Sc., University College; W. F. V. Bonney, Middlesex Hospital; E. B. Bostock, Mason College and General Hospital, Birmingham; Margaret M. Traill Christie, London School of Medicine and Royal Free Hospital; W. E. Dixon, B.Sc., St. Thomas's Hospital; A. R. J. Douglas, St. Bartholomew's Hospital; G. G. Genge, St. Thomas's Hospital; T. H. Hunt, Yorkshire College; C. E. M. Kelly, Owens College; O. M. S. Lewin, London School of Medicine and Royal Free Hospital; C. Oldfield, Yorkshire College: N. H. Pike, Guy's Hospital; G. B. Price, Bristol Medical School and St. Bartholomew's Hospital; C. Roberts, Middlesex Hospital: A. R. H. Skey, St. Bartholomew's Hospital; A. H. Spicer, Guy's Hospital; A. Stanley, M.D., St. Mary's Hospital: F. B. Thornton, St. Thomas's Hospital; E. O. Thurston, St. Thomas's Hospital; F. T. Waldron, London Hospital; Lizzie Wilks, M.D., London School of Medicine for Women; F. K. Wilson, Westminster Hospital.

Clerical, Professional, Commercial, Pocket, and Scribbling

### DIARIES

FOR

### 1897.

In every variety of Size, Style, and Binding,

To be had of all Booksellers and Stationers in Great Britain and Abroad, and at the Railway Bookstalls.

28 & 29, SOUTHAMPTON STREET, STRAND, LONDON, W.C.

---

The Most EFFECTUAL BANDAGE for the CURE of ULCERS and other DISEASES of the LEG.

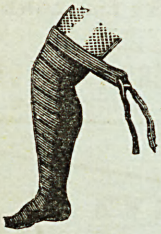

Statham's Porous or Solid Rubber Bandages, with Tapes or Buttons, in Grey or Black, made from the finest Para Rubber.

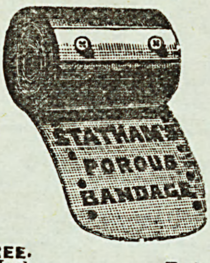

PRICES, POST FREE.

| Feet | Inch. | Each. |
|---|---|---|
| 5 by | 2½ wide | 2s. |
| 7½ by | 2½ „ | 3s. |
| 10½ by | 2½ „ | 4s. |
| 15 by | 2½ „ | 6s. |
| 21 by | 2½ „ | 8s. |

| Feet. | Inch. | Each. |
|---|---|---|
| 5 by | 3 wide | 2s. 3d. |
| 7½ by | 3 „ | 3s. 6d. |
| 10½ by | 3 „ | 4s. 6d. |
| 15 by | 3 „ | 6s. 9d. |
| 21 by | 3 „ | 9s. 0d. |

H. STATHAM & CO., CORPORATION STREET, MANCHESTER.

---

Price 5s. 6d., post free. Gilt edges. Leather. Two Pockets, Pencil Case, and Fastener. Very strong.

### WRIGHT'S VISITING LISTS, 1897.

In Three Forms, A, B, and C.

AN ELEGANT PRESENT

For a Medical Man, with Initials Stamped in Gold.

"The most compendious, most generally useful, and in every respect the best."—*Liverp. Med. Journ.*

JOHN WRIGHT AND CO., PUBLISHERS, BRISTOL.

---

Just Out. Price 1s.

### CHARITY ORGANISATION AND JESUS CHRIST,

By Rev. C. L. MARSON, M.A.

This tractate is a plea for the needy and against the niggardly. It is addressed to such men and women as think that Scientific Almsgiving which conflicts with Scientific Theology, or with Redeemed Humanity, had better get out of the way as soon as possible.

London: THE SCIENTIFIC PRESS (LIM.), 28 & 29, Southampton Street, Strand, W.C.

